# Remaining Useful Life Estimation of Insulated Gate Biploar Transistors (IGBTs) Based on a Novel Volterra k-Nearest Neighbor Optimally Pruned Extreme Learning Machine (VKOPP) Model Using Degradation Data

**DOI:** 10.3390/s17112524

**Published:** 2017-11-03

**Authors:** Zhen Liu, Wenjuan Mei, Xianping Zeng, Chenglin Yang, Xiuyun Zhou

**Affiliations:** 1 School of Automation Engineering, University of Electronic Science and Technology of China, Chengdu 611731, China; 2015070903035@std.uestc.edu.cn (W.M.); yangclin@uestc.edu.cn (C.Y.); zhouxy@uestc.edu.cn (X.Z.); 2 Quanzhou Institute of Equipment Manufacturing, Haixi Institutes, Chinese Academy of Sciences, Quanzhou 362200, China; 201321070144@std.uestc.edu.cn

**Keywords:** remaining useful life, IGBT, prediction model, VKOPP, degradation data

## Abstract

The insulated gate bipolar transistor (IGBT) is a kind of excellent performance switching device used widely in power electronic systems. How to estimate the remaining useful life (RUL) of an IGBT to ensure the safety and reliability of the power electronics system is currently a challenging issue in the field of IGBT reliability. The aim of this paper is to develop a prognostic technique for estimating IGBTs’ RUL. There is a need for an efficient prognostic algorithm that is able to support in-situ decision-making. In this paper, a novel prediction model with a complete structure based on optimally pruned extreme learning machine (OPELM) and Volterra series is proposed to track the IGBT’s degradation trace and estimate its RUL; we refer to this model as Volterra k-nearest neighbor OPELM prediction (VKOPP) model. This model uses the minimum entropy rate method and Volterra series to reconstruct phase space for IGBTs’ ageing samples, and a new weight update algorithm, which can effectively reduce the influence of the outliers and noises, is utilized to establish the VKOPP network; then a combination of the *k*-nearest neighbor method (KNN) and least squares estimation (LSE) method is used to calculate the output weights of OPELM and predict the RUL of the IGBT. The prognostic results show that the proposed approach can predict the RUL of IGBT modules with small error and achieve higher prediction precision and lower time cost than some classic prediction approaches.

## 1. Introduction

As power electronic equipment has come into widespread use, insulated gate bipolar transistor (IGBT) fully controlled power electronic devices, combining the facile drive of MOSFET with the low conduction loss of a bipolar junction transistor (BJT), and possess good switching performance, have found wide application in industrial automotive, traction, and solar inverter areas [1,2,3]. Therefore, knowing whether an IGBT is in a normal state is critical to the safe operation of the system [4]. Generally, the performance of a system may gradually decline as IGBTs contain numerous materials with different coefficients of thermal expansion (CTE) with many interfaces, which can wear out and cause overstress failures [5]. There are two typical ways to solve system safety issues. One is condition monitoring and fault diagnosis for an IGBT. In [6], Choi et al. proposed a condition monitoring method of an IGBT module by online V_CE-ON_ measurement. Another is the failure prediction method based on the estimation of remaining useful life (RUL) of an IGBT, which plays an essential role in power semiconductor reliability. To date, there are two approaches to predict RUL [7]: the physics-of-failure (PoF)-based approach and the data-driven approach.

PoF-based prognostic methods rely on extensive knowledge of IGBT chemistry and failure mechanisms. Since the methods involve excessive physical parameters, the models are usually difficult to build precisely [8]. In contrast, the data-driven approaches require prognostic data that reflect the IGBT degradation behavior derived from ordinarily observed operating parameters such as voltage, current, power, and temperature [9,10] without the need of extensive knowledge on the devices.

In [11], Li et al. aged an IGBT module by a temperature cycling test to obtain on-state voltage and current as deterioration parameters; they proposed a method based on particle filter (PF) theory to predict the RUL of IGBTs under test. However, the training process of the algorithm is time consuming, and the prediction accuracy is not high for cases of small samples. In [12], Thakur et al. proposed a temperature prediction method for IGBT modules based on the auto-regressive moving average (ARMA) model. However, the applicable range of the AR model for temperature prediction is limited to linear time series. Wu et al. [13] established a back propagation (BP) artificial neural network model to predict the junction temperature of an IGBT power module by measuring the device’s saturation voltage and collector current under a specified temperature. However, this paper remains a preliminary study for semiconductor temperature prediction by using neural networks, and the prediction accuracy may decline as the quantity of training data decreases. In [14], Mominul et al. developed a data-driven prognostic approach based on the neural network (NN) and adaptive neuro fuzzy inference system (ANFI) models to predict the degradation of IGBT devices. The predicted RUL matches the actual RUL, and the accuracy improves as the devices complete more degradation phases of the test. However, the results during the early test time phase cannot be accurate. Therefore, although these data-driven methods have useful characteristics such as simple model building and rapid calculation, the accuracy of prediction is difficult to guarantee for nonlinear time series or inadequate samples, especially when the IGBT data contain too little or irrelevant information related to forecasting the track, resulting in inadequate training or over-fitting and damaging the RUL prediction performance of IGBTs.

To remedy these weaknesses of IGBT prediction models, this paper aged the IGBT module by a temperature cycling test to acquire the collector–emitter ON voltage (V_CE_), collector–emitter ON current (I_CE_), and case temperature (T) as the indicator of the RUL and fully utilized the Volterra series model [15,16] and optimally pruned extreme learning machine (OPELM) model [17,18,19,20] to achieve a perfect RUL prediction result of IGBTs.

In [21], the author noted that the Volterra series and single-hidden layer feed forward neural network (SLFN) model are inherently in contact and similar in function, structure and method for solving, in the case of the reconstructed phase space vector as the input vector of the SLFN model. In addition, the original extreme learning machine (ELM) algorithm [17], which uses SLFN with very few steps and very low computational cost, has all the properties of SLFN. In addition, the OPELM inherits the characteristics of ELM and wraps this extended algorithm possessing higher generalization and robustness. Thus, the Volterra series and OPELM model are inherently equivalent. Owing to the equivalency of both models, in this paper we propose a novel prediction model named Volterra k-nearest neighbor adaptive OPELM prediction (VKOPP) model to trace the IGBT’s degradation and estimate its RUL with the superiority of both methods.

The aim of this paper is to develop a prognostic technique using VKOPP model for estimating the RUL of power electronic components. Its structure is as follows: the next section presents the parametric investigation of the IGBT module degradation process. The mathematical description and the basic idea of the VKOPP algorithm are depicted in Section 3. Section 4 demonstrates the specific steps for IGBT RUL prediction based on the VKOPP method. Section 5 gives some illustrative examples to show the working mechanisms of the VKOPP model for some other datasets and results concerning computational speed and accuracy for various prediction methods. Section 6 presents the experimental results and analysis by using our approach to IGBT RUL prediction. Finally, the discussion and conclusion are given in Section 7.

## 2. Aging Experiment

### 2.1. Parametric Investigation of IGBT Module Degradation

The power cycling test is one of the available standard reliability test methods used widely to test the long-term behavior of power devices. However, its results rely on the test parameters. Hence, selection of the most appropriate parameters to monitor is vital to facilitate more accurate aging experiment results [22]. Therefore, it is necessary to study some characteristics of IGBT modules, especially their failure mechanisms. This section focuses on study of some characteristic indexes of IGBT modules in the process of performance degradation and presents a comparison of advantages and disadvantages of several parameters to choose the best parameters to monitor.

**Figure 1:**
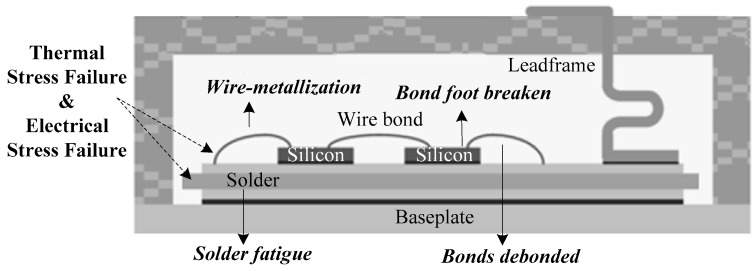
Cross-sectional view of the IGBT module.

As a combination of a MOSFET and a BJT, IGBT has switching characteristics similar to a MOSFET and high current and voltage capabilities similar to a BJT, and thermal stress failure and electrical stress failure are two typical failure mechanisms of it [23,24,25,26,27]. Since the IGBT modules are always under power cycling, the bond foot, solder layer and wire-bonds part, which have close distance with each other in the module, are too weak to sustain the strike of thermal stress and can easily lead to failure (see Figure 1). In particular, cracks can appear because of solder fatigue failure, and with the growth of the cracks, cavities will form in the interior of the IGBT, and even detachment and breakage of the wires can occur, finally causing failure of the IGBT.

In Figure 1, the power module of the IGBT consists of a multi-layer structure. Each layer of material has different cofficients of thermal expansions, which leads to different levels of thermal expansion of the units connected to each other in the interior of the module under thermal stress, causing deformations and typical thermal stress failures such as the peeling and the root breakage of the wire bonds, solder fatigue and wire-metallization. Meanwhile, the IGBT module will sustain extreme over-electrical stress when it is running under over-voltage or over-current, causing local heating effect problems and the corresponding shape changes inside it, which consequently lead to IGBT failure.

Both thermal stress failure and electrical stress failure can lead to the abnormalities in the interior structure of the IGBT module. The abnormalities of the internal structure will lead to changes to the external characteristic parameters [28]. These key parameters such as junction-case thermal resistance [23], gate voltage [24], turn-off time [25], and collector–emitter saturation voltage [26], directly reflect the state of aging of the device. Therefore, these parameters can be used as the monitoring parameters of RUL prediction for the IGBT module. Table 1 shows the relation between the typical failure mechanisms and external characteristic parameters, and Table 2 summarizes the advantages and disadvantages of different IGBT module status monitoring parameters.

As shown in Table 2, junction-case thermal resistance, gate voltage and turn-off time not only require a sensor of high measurement accuracy, but are also vulnerable to the outside influence of other factors. In contrast, the collector–emitter saturation voltage, which is affected by the collector current and case temperature, is relatively simple to acquire, and it can significantly reflect the solder layer fatigue and wire-bond failure. Hence, the precursor parameters for IGBT failure prognostics are identified as collector–emitter ON voltage (V_CE_), collector–emitter ON current (I_CE_), and case temperature (T). These precursor parameters are used for the monitoring parameters of IGBT RUL prediction in the accelerated life test.

### 2.2. IGBT Experimental Data Acquisition

**Figure 2:**
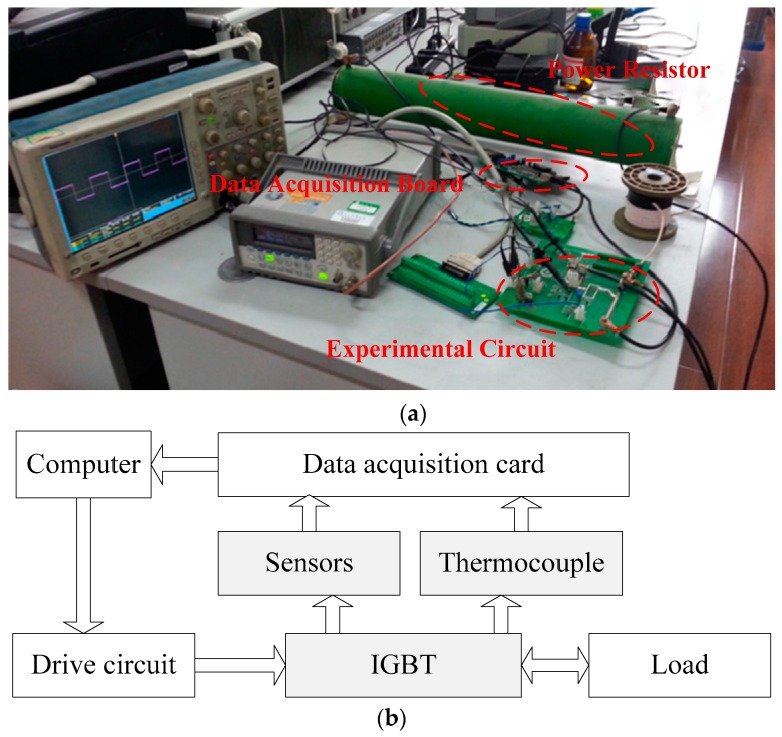
Accelerated life test system: (**a**) Picture of the test system; (**b**) Block diagram of the test system.

**Figure 3:**
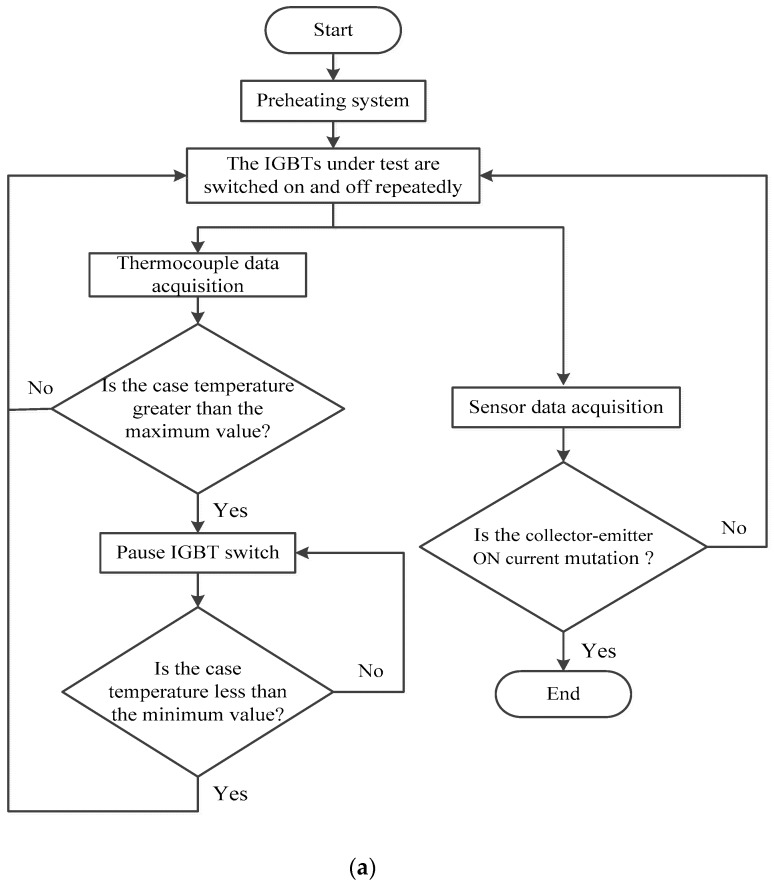

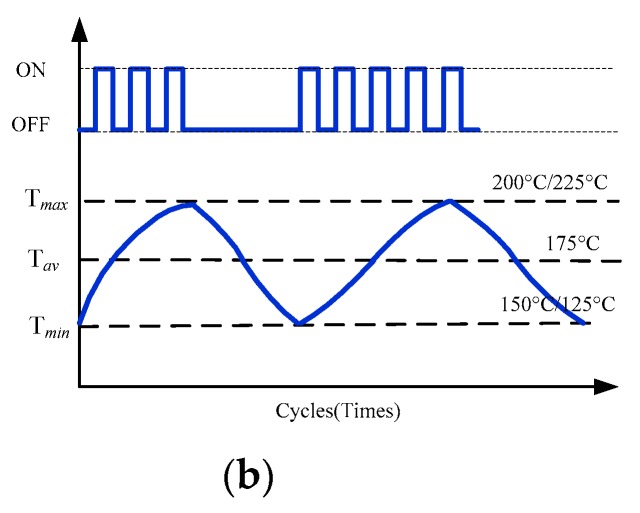
Accelerated life test control process: (**a**) Control flowchart; (**b**) Temperature cycle.

In the IGBT accelerated life test (shown in Figure 2), the IGBT devices (600 V/6 A) were packaged in a TO-220AB package along with a soft recovery diode from International Rectifier (El Segundo, CA, USA). A pulse-width-modulated (PWM) signal with an amplitude of 15 V and a frequency of 1 or 5 kHz was chosen to be the gate signal. Our experimental system mainly consists of the driven circuit module, the voltage and current sensor module, the thermocouple module, the data acquisition module and the computer-control system. The driven circuit module adopts the application specific intergrated circuit IRS21271 driver, to form the driven wave of the IGBT and control the IGBT’s ON and OFF. The current sensor (BJHCS-104 series Hall current sensor) and voltage sensor (HV25-P series Hall voltage sensor) are mainly used for responsing to the change of state of the IGBT and transferring the data from the data acquisition card to the computer. The thermocouple module includes the T type thermocouple and the matched signal conditioning circuit to realize the measurement of the temperature of the surface of the power device, which is the key module in the accelerated life experiment. During the temperature cycling experiment (the control process is shown in Figure 3a,b), the IGBT under testing was switched on and off repeatedly until the case temperature reached the maximum value T_max_, which had been set before. When T_max_ was attained, the device was powered off until the temperature decreased to T_min_. The device was then cycled between the temperatures T_max_ and T_min_ while the average was set to 175 °C with an optional swing. Meanwhile, the precursor parameters V_CE_, I_CE_, and T were in-situ measured and preserved by a data acquisition system until failure of the IGBT under testing, which was observed as a large increase in collector-emitter ON current caused by latch-up [29]. The experiment condition setting is shown in Table 3.

**Figure 4:**
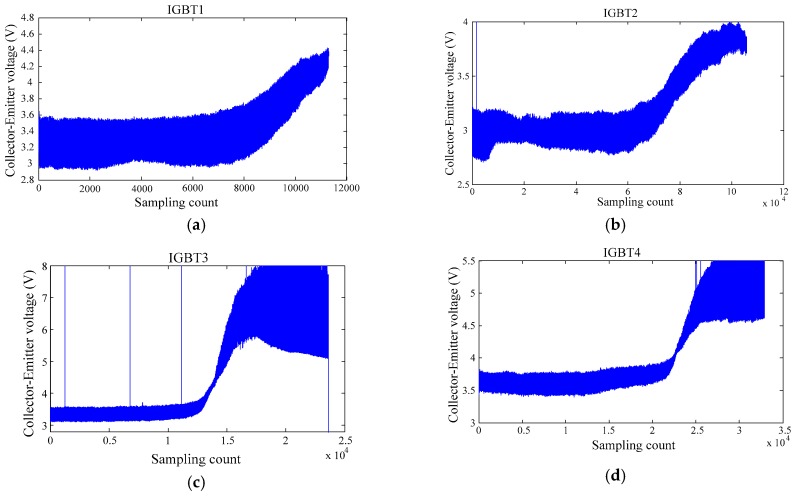
Experimental data. (**a**) IGBT1 expermental raw data; (**b**) IGBT2 expermental raw data; (**c**) IGBT3 expermental raw data; (**d**) IGBT4 expermental raw data.

Figure 4 shows V_CE_ waveforms of IGBT modules during our temperature-cycling test, and these data exhibit a typical and significant degradation trace, which will be used for the development and verification of the VKOPP prediction model.

From the original experimental data of Figure 4, with the gradient failure of the IGBT module, the collector-emitter saturation voltage goes up. This phenomenon is mainly caused by the different cut effects by the thermal stress between different structural materials, which leads to obvious cracks and cavities in the solder layer and linking lead. Both cracks and cavities of the solder layer and linking lead can cause heating effects that accelerate devices and provide positive feedback and seriously increase the odds of the distribution of hotspots, leading to the failure of the measured devices. During the data collection of the whole failure process, the experimental circuit has some disturbances, such as instability of the driving waveform, the transport delay of the twisted pairs, the stray inductance caused by the load network and the error of the PCB circuit board, leading to the collector-emitter saturation voltage of the raw experimental data not being equal to the typical value provided by the device manual and mixed with noisy data and bad points. Therefore, before performing the failure prediction of the IGBT, the raw data need to be preprocessed, including getting rid of bad points, signal denosing, normalization and dimensional reduction. The data pre-processing steps will be introduced in Section 6.2.

## 3. Data Transformation Based on the Phase Space Reconstruction

Here, we use the delay-coordinate method [30,31] to reconstruct the phase space of time series $\left\{x_{t},t=1,2,\cdots ,n\right\}$ of the IGBT degradation process. Points in the phase space are expressed as $x_{t}=[x(t),x(t-\tau ),\cdots ,x{\left(t-(m-1)\tau \right]}^{T}$, where *m* is an embedding dimension and *τ* is a time delay, and we use the minimal differential entropy ratio(ER) [32] to optimize *m* and *τ* simultaneously in place of the inconsistency of mutual information method and false-nearest-neighbors method. The optimization process is as follows.

**Figure 5:**
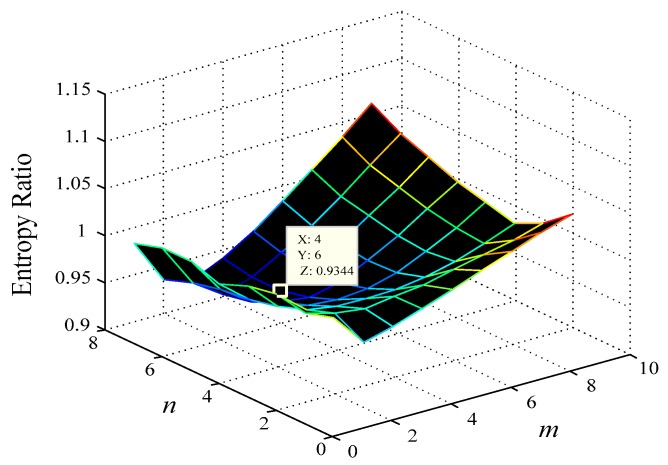
The IGBT experimental data (IGBT4) entropy ratio diagram.

The substitution data of the given signal are $x_{s,i}(t),i=1,2,\cdots ,N_{S}$, where $N_{S}$ is the number of $x_{s,i}(t)$. The entropy ratio $R_{ent}$ is defined as follows:

(1) $$R_{ent}(m,\tau )=I(m,\tau )(1+\frac{m\ln(N)}{N})$$

(2) $$I(m,\tau )=\frac{H(x,m,\tau )}{<H(x_{s,i},m,\tau )>}$$

(3) $$H(x)=\sum_{j=1}^{N}\ln(N\rho_{j})+\ln2+C_{E}$$

where N(N=n−(m−1)τ) is the number of delay vectors, and ${<•>}_{i}$ is the average computation operator that calculates the $N_{S}$-alternative data ER $H(x_{s,i})\;(i=1,2,\cdots ,N_{S})$ average. $\rho_{j}$ is the Euclidean distance between the j-th delay vector and its nearest neighbor points. Euler’s constant *C*_E_ ≈ 0.5772.

m and τ are then changed within a certain range, and the ER diagram is drawn, where the minimum values on the ER graph in the m axis and τ axis correspond to the optimal embedding dimension $m_{opt}$ and delay time $\tau_{opt}$; e.g., for IGBT4 experimental data, its ER diagram is shown in Figure 5.

Thus, it can be embedded into the reconstructed delay phase space with $m_{opt}$ and $\tau_{opt}$, and a point in the reconstructed phase space can be expressed as:

(4) $$x_{t}=[x(t),x(t-\tau_{opt}),\cdots ,x{\left(t-(m_{opt}-1)\tau_{opt}\right]}^{T}\;(t=1,2,\cdots ,n)$$

Assuming the input of the nonlinear discrete dynamical system as Equation (4) and the output as y(t), the discretization Volterra series model can be expressed by Equation (5):

(5) $$\begin{array}{lll} y(t) & = & \sum_{p}\sum_{l_{1},\cdots ,l_{p}=0}^{m_{opt}-1}h_{p}(l_{1},\cdots ,l_{p})x(t-l_{1}\tau_{opt})x(t-l_{2}\tau_{opt})\cdots x(t-l_{p}\tau_{opt})\\ & = & h_{0}(l_{0})+\sum_{l_{1}=0}^{m_{opt}-1}h_{p}(l_{1})x(t-l_{1}\tau_{opt})+\sum_{l_{1}=0}^{m_{opt}-1}\sum_{l_{2}=0}^{m_{opt}-1}h_{2}(l_{1},l_{2})x(t-l_{1}\tau_{opt})x(t-l_{2}\tau_{opt})\\ & & +\sum_{l_{1}=0}^{m_{opt}-1}\sum_{l_{2}=0}^{m_{opt}-1}\sum_{l_{3}=0}^{m_{opt}-1}h_{3}(l_{1},l_{2},l_{3})x(t-l_{1}\tau_{opt})x(t-l_{2}\tau_{opt})x(t-l_{3}\tau_{opt})\\ & & +\cdots \\ & & +\sum_{l_{1}=0}^{m_{opt}-1}\sum_{l_{2}=0}^{m_{opt}-1}\sum_{l_{3}=0}^{m_{opt}-1}\cdots \sum_{l_{p}=0}^{m_{opt}-1}h_{p}(l_{1},l_{2},l_{3},\cdots ,l_{p})x(t-l_{1}\tau_{opt})x(t-l_{2}\tau_{opt})x(t-l_{3}\tau_{opt})\cdots x(t-l_{p}\tau_{opt}) \end{array}$$

where $h_{p}(l_{1},\cdots ,l_{p})\;(p=1,2,\cdots )$ denotes the system’s p-th order Volterra kernel.

Because the Volterra filter is a nonlinear adaptive FIR filter, for *p*-order Volterra series, the filter coefficient vector and the input signal vector are given as follows:

(6) $$\begin{array}{ll} H^{\prime }(t)= & [h_{0}^{\prime },h_{1}^{\prime }(0),h_{1}^{\prime }(1),\cdots ,h_{1}^{\prime }(m_{opt}-1),h_{2}^{\prime }(0,0),h_{2}^{\prime }(0,1),h_{2}^{\prime }(1,0),\cdots ,h_{2}^{\prime }(m_{opt}-1,m_{opt}-1),\\ & \cdots ,h_{p}^{\prime }(0,0,\cdots ,0),h_{p}^{\prime }(0,1,\cdots ,0),h_{p}^{\prime }(1,0,\cdots ,0)，\cdots ,h_{p}^{\prime }(m_{opt}-1,m_{opt}-1,\cdots ,m_{opt}-1){]}^{T} \end{array}$$

(7) $$\begin{array}{ll} Z^{\prime }(t)= & [1,x(t),x(t-\tau_{opt}),\cdots ,x(t-(m_{opt}-1)\tau_{opt}),x^{2}(t),x(t)x(t-\tau_{opt}),x(t-\tau_{opt})x(t),\\ & \cdots ,x^{2}(t-(m_{opt}-1)\tau_{opt}),\cdots ,x^{p}(t),x(t)x(t-\tau_{opt})x^{p-2}(t),x(t-\tau_{opt})x^{p-1}(t),\cdots ,x^{p}(t-(m_{opt}-1)\tau_{opt}){]}^{T} \end{array}$$

where the vector dimensions of $H^{\prime }(t)$ and $Z^{\prime }(t)$ are both $1+m_{opt}+m_{opt}^{2}+\cdots +m_{opt}^{p}$.

Combining similar terms, and making:

(8) $$\left\{ \begin{array}{l} h_{0}=h_{0}^{\prime };\\ h_{1}(i)=h_{1}^{\prime }(i);\\ h_{2}(i,j)=h_{2}^{\prime }(i,j)+h_{2}^{\prime }(j,i);\\ h_{3}(i,j,k)=h_{3}^{\prime }(i,j,k)+h_{3}^{\prime }(i,k,j)+h_{3}^{\prime }(j,i,k)+h_{3}^{\prime }(j,k,i)+h_{3}^{\prime }(k,i,j)+h_{3}^{\prime }(k,j,i);\\ \cdots \end{array}\right.$$

$i,j,k\in \{0,1,\cdots ,m_{opt}-1\}$, and i≤j≤k, then Equations (6) and (7) can be written as:

(9) $$\begin{array}{ll} H(t)= & [h_{0},h_{1}(0),h_{1}(1),\cdots ,h_{1}(m_{opt}-1),h_{2}(0,0),h_{2}(0,1),\cdots ,h_{2}(m_{opt}-1,m_{opt}-1),\\ & \cdots ,h_{p}(0,0,\cdots ,0),h_{p}(0,0,\cdots ,1),\cdots ,h_{p}(m_{opt}-1,m_{opt}-1,\cdots ,m_{opt}-1){]}^{T} \end{array}$$

(10) $$\begin{array}{ll} Z(t)= & [1,x(t),x(t-\tau_{opt}),\cdots ,x(t-(m_{opt}-1)\tau_{opt}),x^{2}(t),x(t)x(t-\tau_{opt}),\cdots ,x^{2}(t-(m_{opt}-1)\tau_{opt}),\\ & \cdots ,x^{p}(t),x^{p-1}(t)x(t-\tau_{opt}),\cdots ,x^{p}(t-(m_{opt}-1)\tau_{opt}){]}^{T} \end{array}$$

where the vector dimensions of H(t) and Z(t) are both $1+m_{opt}+\sum_{i=0}^{m_{opt}-1}C_{m_{opt}-i}^{1}+\cdots +\sum_{i=-p+1}^{m_{opt}-p}C_{m_{opt}-i}^{p}$.

Thus, Equation (5) can be simplified as $y(t)={\left(Z^{\prime }(t)\right)}^{T}(H^{\prime }(t))=$ $Z^{T}(t)H(t)\;(t=1,2,\cdots ,n)$. In practice, the truncation order is generally one-order truncation, second-order truncation or third order intercept. Hereinafter, for the sake of solving the higher order kernel estimates of Volterra series, we pre-treat the IGBT experiment data to obtain Z(t) as the input of the VKOPP model.

## 4. Developing the VKOPP Model

**Figure 6:**
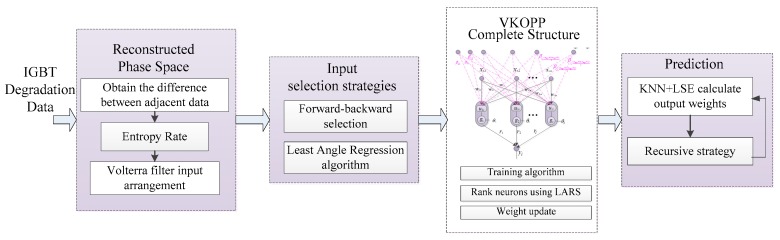
Prediction principles of the proposed VKOPP model.

The relations between V_CE_, I_CE_ and T are nonlinear and complex, while the Volterra series demonstrates great appeal because its output is the linear function of the filter core. Therefore, the existing linear tools are useful to analyze the filtering performance. Based on the equivalence of the Volterra series and OPELM model [18], the VKOPP model was established to trace the IGBT degradation by using both methods. The prediction principle is shown in Figure 6.

### 4.1. VKOPP Complete Structure

**Figure 7:**
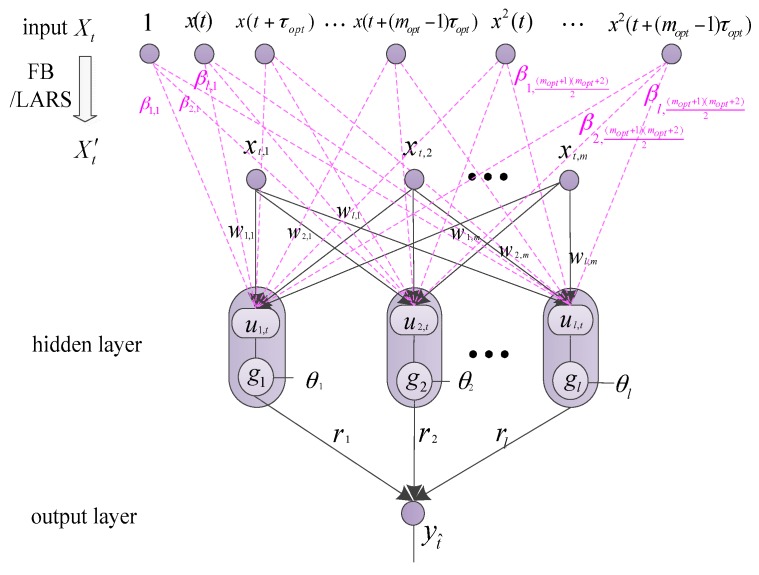
VKOPP complete structure.

Figure 7 shows the complete structure of the VKOPP model. In Figure 7, *Z*(*t*), which is described in Section 3, is used as the input vector $X_{t}$ at moment *t* of the VKOPP model

(11) $$\begin{array}{ll} X_{t}= & [1,x(t),x(t+\tau_{opt}),\cdots ,x(t+(m_{opt}-1)\tau_{opt}),x^{2}(t),x(t)x(t+\tau_{opt}),\cdots ,x^{2}(t+(m_{opt}-1),\\ & \cdots ,x^{p}(t),x^{p-1}(t)x(t+\tau_{opt}),\cdots ,x^{p}(t+(m_{opt}-1)\tau_{opt}){]}^{T}\;(t=1,2,\cdots ,n) \end{array}$$

where the vector dimension of $X_{t}$ is $1+m_{opt}+\sum_{i=0}^{m_{opt}-1}C_{m_{opt}-i}^{1}+\cdots +\sum_{i=-p+1}^{m_{opt}-p}C_{m_{opt}-i}^{p}$. The training expected output is $T=(y_{1},y_{2},\cdots ,y_{t},\cdots ,y_{n})$, with $y_{t}=x(t+m_{opt}\tau_{opt})\;(t=1,2,\cdots ,n)$.

When the input selection strategy—i.e., the forward-backward algorithm (FB) [33] or least angle regression algorithm (LARS) [34], is used, the input vector of hidden units in the VKOPP model can be expressed as:

(12) $$X_{t}^{\prime }={\left[1,x\left(t+c_{1}\tau_{opt}\right),x\left(t+c_{2}\tau_{opt}\right),\cdots ,x\left(t+c_{b}\tau_{opt}\right),\cdots ,x\left(t+c_{e}\tau_{opt}\right)x\left(t+c_{f}\tau_{opt}\right),\cdots \right]}^{T}$$

where $c_{b},c_{e},c_{f}\in \left\{0,1,\cdots ,m_{opt}-1\right\},$ with $c_{e}\leq c_{f}$. The vector dimensions of $X_{t}^{\prime }$ are denoted as *m*, and then $X_{t}^{\prime }$ can be simplified as $X_{t}^{\prime }={\left[x_{t,1},x_{t,2},\ldots ,x_{t,m}\right]}^{T}\;(t=1,2,\cdots ,n)$.

In Figure 7, $g_{s}\;(s=1,2,\cdots ,N)$ is the activation function using a combination of three different types of kernels—linear, sigmoid and Gaussian—for robustness and improvement of generality. *N* is the hidden neurons; $w={\left(w_{s,i}\right)}_{N\times m}$ and $r=(r_{s})$ are the input weights and output weights of the VKOPP model, respectively; and $\theta_{s}$ is the biases. At moment t, the input of the s (s=1,2,⋯,N) hidden unit is then $H_{s,t}=g_{s}(u_{s,t}-\theta_{s}),u_{s,t}=\overset{m}{\underset{i=0}{\sum }}w_{s,i}x_{t,i}$.

Further, it is assumed that via effective pruning of irrelevant variables and training via the OPELM algorithm [18], the actual best number of neurons for the model is l, and the OPELM hidden-layer output matrix is:

(13) $$H=[h_{1},h_{2},\cdots ,h_{j},\cdots ,h_{D}]$$

### 4.2. Training for VKOPP Complete Structure

#### 4.2.1. Original OPELM Algorithm

The OPELM algorithm [18] inherits the characteristics of the ELM [17] and wraps this extended algorithm possessing higher generalization and robustness. The basic principle of OPELM algorithm can be described as follows: first, the ELM model should be constructed. The initial number of hidden nodes is denoted as *N*. After ranking the best neurons using multiresponse sparse regression (MRSR) [18], the target is the network actual output *Y*, while regression matrixes considered by the MRSR are the outputs of the hidden layer kernel functions $H_{i}\;(i=1,2,\cdots ,N)$. Because of the exact ranking provided by MRSR, it is used to rank the neurons of the ELM model. In addition, MRSR is mainly an extension of the least angle regression (LARS) algorithm [34], and when the dimension of the target function is one, the MRSR algorithm is equivalent to the LARS algorithm. *N* hidden layer nodes after sorting are denoted as $\left\{H_{j_{1}}^{1},H_{j_{2}}^{2},\cdots ,H_{j_{i}}^{i},H_{j_{i+1}}^{i+1},\cdots ,H_{j_{N}}^{N}\right\}$, where subscript $1\leq j_{i}\leq N$ and superscript 1≤i≤N represent the serial number of hidden layer nodes before and after sorting, respectively.

Next, the selection of the final model structure is achieved through leave-one-out (LOO) validation:

(14) $$\varepsilon_{i}^{press}=\frac{Y-H_{i}{\bar{\beta }}_{i}}{1-H_{i}pH_{i}^{T}}$$

where *i* represents the *i*-th hidden layer node, $H_{i}$ is the columns of the hidden-layer output matrix after sorting $\bar{H}$, ${\bar{\beta }}_{i}$ is the output weights, and p is defined as $p={\left({\bar{H}}^{T}\bar{H}\right)}^{-1}$. The appropriate number of hidden neurons for the model can then be selected by evaluating the LOO error versus the number of neurons used. The number of hidden neurons after sorting is denoted as *l*. Then, $l=\underset{j\in \{1,\cdots ,N\}}{\arg\min}\sum_{i=1}^{j}\varepsilon_{i}^{press}$. It can be noted that with the MRSR ranking step, the convergence is faster, while the number of neurons is far fewer, leading to a sparser network with the same performance.

#### 4.2.2. VKOPP Training Algorithm

Desspite that fact that the OPELM algorithm is obtained with very few steps and very low computational cost, there are still some critical issues to be solved. The major problem is that the output layer weight estimation results will be very poor if there are collinearity or gross errors in the training data, so in this section, we propose a method based on the least squares method weighted by M estimation to obtain the output weights and output matrix; we refer to this new training algorithm as the VKOPP algorithm, which uses M estimation to improve the robustness with the weighted least squares method to calculate the regression coefficients and to obtain each output weight by the regression residual.

For the training set $\left\{(X_{t},Y_{t})\right\}$ formed by D groups of data, where $X_{t}={\left[x_{t,1},x_{t,2},\cdots ,x_{t,m}\right]}^{T}$ is the input vector and $Y_{t}={\left[y_{t,1},y_{t,2},\cdots ,y_{t,n}\right]}^{T}$ is the corresponding expected output, where *m* and *n* are dimensions of the input samples and output samples. The mathematic expression of the OPELM model can be represented as:

(15) $$Y=\hat{H}\hat{\beta }+e$$

where $\hat{H}$ is the output matrix of the network hidden layer after pruning, $\hat{\beta }$ is the unknown output weight parameter, and *e* is represented as the regression residuals.

This method chooses different types of the influence functions contrapuntally in place of the quadratic sum of residuals in least square method. Here is the chosen influence function proposed in [35]:

(16) $$\rho (e)=\{\begin{array}{ll} \frac{e^{2}}{2} & ,\;\left|e\right|\leq k\\ k\left|e\right|-\frac{k^{2}}{2} & ,\;\left|e\right|>k \end{array}$$

where *k* is the harmonic constant, with the typical value *k* = 1.3450.

Assume φ(e) as the differential coefficient of ρ(e), which can be represented as:

$$\varphi (e)=\frac{d\rho (e)}{de}=\{\begin{array}{cc} -k, & e<-k\\ e, & \left|e\right|\leq k\\ k, & e>k \end{array}$$

For the OPELM model, the optimization objective function of the regression residuals is:

(17) $$Q(\hat{\beta })=\overset{D}{\underset{t=1}{\sum }}\rho (e_{t})=\overset{D}{\underset{t=1}{\sum }}\rho (Y_{t}-{\hat{H}}_{t}\hat{\beta })$$

We calculate the partial derivative of the output weight parameter $\hat{\beta }$ and make the partial derivative equal to zero, that is:

(18) $$\overset{D}{\underset{t=1}{\sum }}\varphi (Y_{t}-{\hat{H}}_{t}\hat{\beta }){\hat{H}}_{t}=0$$

where ${\hat{H}}_{t}=\left[\begin{array}{ccc} g({\hat{w}}_{1,t}^{T}X_{t}-{\hat{\theta }}_{1}) & \cdots & g({\hat{w}}_{l,t}^{T}X_{t}-{\hat{\theta }}_{l}) \end{array}\right]$, $\hat{\beta }={\left[{\hat{\beta }}_{1},\cdots ,{\hat{\beta }}_{l}\right]}^{T}$, and $e_{t}$ is the residual of the *t* sample.

In M estimation, confirm the weight of each output weight parameters $\hat{\beta }$ by the regression residuals. In other words, give a large proportion of weight to the output weight parameters $\hat{\beta }$ with low regression residuals. To standardize the regression residuals the scale estimation factor *S* is introduced to the weight function, which generally valued as the median absolute deviation (MAD) divided by the constant 0.6745. Therefore, the new OPELM model can be expressed as:

(19) $$Y=\hat{H}\hat{\beta }+v$$

where, $v=\frac{e}{s}=\frac{0.6745e}{\mathrm{med}(\left|e\right|)}$, with med represented as the median calculation.

From the Equation (18), we can get:

(20) $$\overset{D}{\underset{t=1}{\sum }}\varphi \left(Y_{t}-{\hat{H}}_{t}\hat{\beta }\right){\hat{H}}_{t}=\sum_{t=1}^{D}\varphi (v_{t}){\hat{H}}_{t}=\sum_{t=1}^{D}\frac{\varphi (v_{t})}{v_{t}}v_{t}{\hat{H}}_{t}=\sum_{t=1}^{D}\gamma_{t}v_{t}{\hat{H}}_{t}=0$$

Namely:

(21) $${\hat{H}}^{T}\gamma v=0$$

From the Equations (19) and (21), obtain the output weights $\hat{\beta }$ after the OPELM is pruned:

(22) $$Y=\hat{H}\hat{\beta }+v\Leftrightarrow {\hat{H}}^{T}\gamma Y={\hat{H}}^{T}\gamma \hat{H}\hat{\beta }+{\hat{H}}^{T}\gamma v\overset{{\hat{H}}^{T}\gamma v=0}{\Leftrightarrow }{\hat{H}}^{T}\gamma Y={\hat{H}}^{T}\gamma \hat{H}\hat{\beta }\Leftrightarrow \hat{\beta }={\left({\hat{H}}^{T}\gamma \hat{H}\right)}^{-1}{\hat{H}}^{T}\gamma Y$$

The training algorithm of VKOPP is as follows:

**Step** **1.** The number of training set as D, construct the ELM models with N as the number of neurons. Randomly assign the input weights $w_{i}$ and bias of hidden layer $\theta_{i}$. Record the output matrix of hidden layers as ***H*** and the output weight matrix as β.
**Step** **2.** Rank nodes of hidden layers by the MRSR algorithm [21] as $\left\{H_{j_{1}}^{1},H_{j_{2}}^{2},\cdots ,H_{j_{i}}^{i},H_{j_{i+1}}^{i+1},\cdots ,H_{j_{N}}^{N}\right\}$, where subscript $1\leq j_{i}\leq N$ and superscript 1≤i≤N represent the serial number of hidden layer nodes before and after sorting.
**Step** **3.** Select the optimized number of neurons by the LOO method based on the ranked order.
**Step** **4.** Update the input weights $w_{i}$ and threshold parameter $\theta_{i}$ after pruning. Calculate the output matrix of the hidden layer $\hat{H}$ further.
**Step** **5.** Use the output matrix ${\hat{\beta }}^{\left(0\right)}={\hat{H}}^{+}Y={\left({\hat{H}}^{T}\hat{H}\right)}^{-1}{\hat{H}}^{T}Y$ from the least square estimation of the traditional OPELM to access the initial regression residual $e_{0}$ and standardize $e_{0}$ as v.
**Step** **6.** Obtain the initial weight of the t(t=1,2,⋯,D) training samples by $\gamma_{t}=\frac{\varphi (v_{t})}{v_{t}}$.
**Step** **7.** Use ${\hat{\beta }}^{\left(1\right)}$ of Equations (22) instead of the ${\hat{\beta }}^{\left(0\right)}$ to achieve the new regression residual $e_{1}$, and the new weights of the output weight matrix of each training samples based on the new regression residual.
**Step** **8.** Return to step 6, and so on, calculate the output weight parameter $\hat{\beta }$. Continue the iteration until the absolute value of the differences between the estimated values of two adjacent steps meet up with the given standard error, that is $\max(\left|{\hat{\beta }}^{\left(i\right)}-{\hat{\beta }}^{\left(i-1\right)}\right|)<\xi$.

### 4.3. Network Output Prediction

In the output phase of the VKOPP model, a combination of the *k*-nearest neighbor method (KNN) [36] and least squares estimation (LSE) method [37] is used to calculate the output weights of OPELM and predict the RUL of IGBT. This new weight update method can effectively reduce the influence of the outliers and noises, leading to improve the accuracy of OPELM algorithm.

Assuming that the number of sample data is *D* and several nearest vectors from matrix ***H*** are found to form a new matrix by the KNN method. The corresponding output weights are then calculated using the LSE method. The calculation process is as follows:

**Step** **1.** As shown in Figure 7, at this moment, $t=D+1-m_{opt}\tau_{opt}$, the initial input vector of the VKOPP model is $X_{t}$. After performing the input selection strategy, the input is denoted as $X_{t}^{\prime }\;(t=D+1-m_{opt}\tau_{opt})$. The hidden layer output matrix $h_{i}=g_{s}\left(\overset{m}{\underset{i=1}{\sum }}w_{s,i}x_{t,i}-\theta_{s}\right)$ is then calculated.
**Step** **2.** Calculate the Euclidean distance between $h_{i}$ and each vector of the matrix of Equation (13); that is:
  (23) $$S=mean{\left(ones(1,D)\times h_{i}-H\right)}^{2}$$
**Step** **3.** Sequence all distances in S, and find the *l* + 10 nearest neighbor from H of Equation (13) to form a new hidden-layer output matrix $H_{i}$ and the corresponding expected output $Y_{i}$, to obtain the output weights:
  (24) $$r=\{r_{1},r_{2},\cdots ,r_{l}\}=H_{i}^{+}Y_{i}={\left(H_{i}^{T}H_{i}\right)}^{-1}H_{i}^{T}Y_{i}$$
  The predicted value of the VKOPP model can then be presented as:
  (25) $$y_{\hat{t}}=f\left(X_{t}^{\prime }\right)=\overset{l}{\underset{s=1}{\sum }}r_{s}g_{s}\left(u_{s,t}-\theta_{s}\right)=\overset{l}{\underset{s=1}{\sum }}r_{s}g_{s}\left(\overset{m}{\underset{i=1}{\sum }}w_{s,i}x_{t,i}-\theta_{s}\right)$$
  In practice, multistep data can also be predicted at a time (i.e., data at moment $\hat{t}+z,z=1,2,\cdots$) by taking the predicted values $y_{\hat{t}}$ as known data to predict the next ones and continue the process. Hence, the value for the future q moments is then predicted in the following form:
  $$\begin{array}{ccc} next\;moment & VKOPP\;model\;input & output(predicted\;value)\\ 1 & x_{t,1},x_{t,2},\ldots ,x_{t,m-1},x_{t,m} & y_{\hat{t}}\\ 2 & x_{t,2},x_{t,3},\ldots ,x_{t,m},y_{\hat{t}} & y_{\hat{t}+1}\\ \cdots & \cdots \;\cdots & \cdots \\ q & x_{t,q},x_{t,q+1},\ldots ,y_{\hat{t}+q-3},y_{\hat{t}+q-2} & y_{\hat{t}+q-1} \end{array}$$
  That is, we obtain the *q-step-ahead* (q≥1) predicted value:
  (26) $$y_{\hat{t}+q}=\{\begin{array}{ll} f(x_{t,q+1},\cdots ,x_{m},y_{\hat{t}},\cdots ,y_{\hat{t}+q-1}) & q\in \{1,2,\cdots ,m-1\};\\ f(y_{\hat{t}+q-m},\cdots ,y_{\hat{t}+q-1}) & q\in \{m,m+1,\cdots \}. \end{array}$$
**Step** **4.** At each next *one-step-ahead* (or *q-step-ahead*) prediction, update $h_{i}$ and γ; then, calculate the predicted value.

## 5. VKOPP Model-Based IGBT’s RUL Prediction

In this section, the proposed VKOPP model is applied to predict the IGBT RUL, and the specific steps are as follows:

**Step** **1.** Pre-treat the IGBT degradation data: the original dataset is normalized as DS={x(1),x(2),⋯,x(D)}, where D is the number of sample data. Take the difference between adjacent data as the input, and then obtain new dataset DN={0,x(2)−x(1),⋯x(i+1)−x(i),⋯,x(D)−x(D−1)} and mark it as DN={∇x(1),∇x(2),⋯,∇x(D)}.
**Step** **2.** Adopt the minimal differential entropy ratio method to optimize embedding dimension d and delay time *τ* on dataset DN at the same time. Map the data to the *d*-dimensional feature space by using the windowize function in Matlab to obtain the input vector $\left\{X_{1},X_{2},\cdots ,X_{t},\cdots ,X_{n}\right\}$ (n=D−dτ), where $X_{t}=\{\nabla x(t),\nabla x(t+\tau ),\cdots ,\nabla x(t+(d-1)\tau )\}\;(t=1,2,\cdots ,n)$. To facilitate the calculations, a two-order truncated discretization Volterra model is taken as an example in the following. Thus, the input vector can be expressed as:
  $$\begin{array}{c} X_{t}=\{1,\nabla x(t),\nabla x(t+\tau ),\cdots ,\nabla x(t+(d-1)\tau ),\\ \nabla x^{2}(t),\nabla x(t)\nabla x(t+\tau ),\cdots ,\nabla x^{2}(t+(d-1)\tau )\} \end{array}$$
  where the vector dimensions of ***X_t_*** is (*d*+1)(*d*+2)/2. The training expected output is $Y=\{\nabla y_{1},\nabla y_{2},\cdots ,\nabla y_{t},\cdots ,\nabla y_{n}\}$, with $\nabla y_{t}=\nabla x(t+d\tau )$.
**Step** **3.** When the input selection strategy (i.e., FB or LARS) is used, the input vector of hidden units can be expressed as $X_{t}^{\prime }=\{1,\nabla x\left(t+c_{1}\tau \right),\nabla x\left(t+c_{2}\tau \right),\cdots ,\nabla x\left(t+c_{b}\tau \right),\cdots ,\nabla x\left(t+c_{e}\tau \right)\nabla x\left(t+c_{f}\tau \right),\cdots \}$. where $c_{b},c_{e},c_{f}\in \left\{0,1,\cdots ,d-1\right\}$, with $c_{e}\leq c_{f}$. Suppose the vector dimension of $X_{t}^{\prime }$ is denoted as m; then, $X_{t}^{\prime }$ can be simplified as $X_{t}^{\prime }={\left[x_{t,1},x_{t,2},\cdots ,x_{t,m}\right]}^{T}(t=1,2,\cdots ,n)$.
**Step** **4.** Construct an ELM model with N hidden neurons, and N<D. Take $X_{t}^{\prime }$ obtained by Step3 as the input vector, with the input weights $w={\left(w_{s,i}\right)}_{N\times m}$ and biases of the ELM model $\theta =\theta_{s}$ (s=1,2,⋯,N;i=1,2,⋯,m). At moment t, the input of the s(s=1,2,⋯,N) hidden unit is $u_{s,t}-\theta_{s}=\overset{m}{\underset{i=1}{\sum }}w_{s,i}x_{t,i}-\theta_{s}$, which falls within the interval [−*a*, *a*] (the effective interval of Taylor expansion; if the activation function is different, the interval will be different, and the default is [−1, 1]). Withal, the input weights w and biases θ are initialized randomly in the interval [−a/m,a/m] while satisfying $\theta_{s}=0$ when $u_{s,t}-\theta_{s}$ is not within the interval [−*a*, *a*].
**Step** **5.** Rank neurons by using the MRSR algorithm; the N hidden-layer nodes via ranking can be expressed as $\left\{g_{j_{1}}^{1},g_{j_{2}}^{2},\cdots ,g_{j_{i}}^{i},g_{j_{i+1}}^{i+1},\cdots ,g_{j_{N}}^{N}\right\}$, where subscript $1\leq j_{i}\leq N$ and superscript 1≤i≤N represent the serial number of hidden layer nodes before and after sorting, respectively. Further, we select the optimal number of neurons by LOO for the model as l.
**Step** **6.** Update the input weights and the biases of remaining hidden neurons as $w={\left(w_{s,i}\right)}_{l\times m}$ and $\theta =\theta_{s}(s=1,2,\cdots ,l;i=1,2,\cdots ,m)$, respectively; then, compute the OPELM hidden-layer output matrix $H=[h_{1},h_{2},\cdots ,h_{j},\cdots ,h_{D}]$.
**Step** **7.** Utilize the KNN and LSE methods to calculate the output weights of OPELM and prediction. The process is as follows:
  (1) As shown in Figure 7, to predict x(D+1), the initial input vector of the VKOPP model is $X_{t}$ according to Step2. After performing the input selection strategy, the input is denoted as $X_{t}^{\prime }\;(t=D+1-d\tau )$. Then, calculate the hidden-layer output matrix $h_{i}=g_{s}\left(\overset{m}{\underset{i=1}{\sum }}w_{s,i}x_{t,i}-\theta_{s}\right)$.
  (2) Calculate the Euclidean distance between $h_{i}$ and each vector of the matrix in Step 6; that is, $S=mean{\left(ones(1,D)\times h_{i}-H\right)}^{2}$.
  (3) Sequence all distances in S, and find the *l* + 10 nearest neighbor from H in Step 6 to form a new hidden-layer output matrix $H_{i}$ and the corresponding expected output $Y_{i}$, to obtain the output weights $\gamma =\{\gamma_{1},\gamma_{2},\cdots ,\gamma_{l}\}=H_{i}^{+}Y_{i}={\left(H_{i}^{T}H_{i}\right)}^{-1}H_{i}^{T}Y_{i}$.
  The predicted value of VKOPP model can then be presented as:
  (27) $$y_{\hat{t}}=x(D)+\nabla y_{\hat{t}}=x(D)+f\left(x_{t,1},\cdots ,x_{t,m}\right)=x(D)+\overset{l}{\underset{s=1}{\sum }}\gamma_{s}g_{s}\left(\overset{m}{\underset{i=1}{\sum }}w_{s,i}x_{t,i}-\theta_{s}\right)$$
  Further, obtain the *q*-step-ahead (q≥1) predicted value:
  $$y_{\hat{t}+q}=\{\begin{array}{ll} y_{\hat{t}+q-1}+f(x_{t,q+1},\cdots ,x_{m},\nabla y_{\hat{t}},\cdots ,\nabla y_{\hat{t}+q-1}) & q\in \{1,2,\cdots ,m-1\};\\ y_{\hat{t}+q-1}+f(\nabla y_{\hat{t}+q-m},\cdots ,\nabla y_{\hat{t}+q-1}) & q\in \{m,m+1,\cdots \}. \end{array}$$
  At each next one-step-ahead (or *q*-step-ahead) prediction, update *h_i_* and γ, and then calculate the predicted value.
**Step** **8.** The metabolism processing technology [38] is employed to update the training data until the predictive value exceeds the IGBT acceptable performance threshold. Once the prediction is completed, obtain the IGBT RUL prediction results, and exit the program.

## 6. Experimental Results and Analysis of IGBT RUL Prediction

### 6.1. Algorithm Performance Validation and Assessment

Before using the proposed VKOPP algorithm to predict the IGBT RUL, in this section, this algorithm is compared with the original OPELM, Volterra and other typical machine learning algorithms to verify the validity, feasibility, and generalization. In addition, eight different datasets have been chosen for the experiments.

#### 6.1.1. Datasets

Different types of datasets are used, including the simulation data and the actual chaotic time series to test the effectiveness of the VKOPP model. The simulation sequence is Mackey–Glass data (MG) [39] and the actual sequences are laser, daily minimum temperatures (DMT), electricity demand (ED), CATS benchmark (CATS_B) and the sunspot number (SN) [40,41]. In addition, the degradation model in reference [42] is used to generate a set of data that is used to validate the method.

Singular points are sometimes generated by failures, which can be detected by using wavelets in the training section but are unlikely to be predicted from the forecast data by reducing the impact of the singular point for prediction as far as possible. Hence, the MG_S dataset with some unclear singular points in the simulation sequence (i.e., MG) is also used in the experiments.

These different types of datasets have all been processed in the same way: for each dataset, two-thirds are used for the training set, and the remaining one-third is used for the test set. The training sets are then normalized (zero-mean and unit variance), and the test sets are normalized using the same normalization factors as the corresponding training set.

#### 6.1.2. Experiments

For the sake of measuring the prediction performance of the different types of datasets, the mean square error (MSE) and normalized root mean square error (NRMSE) are used as the performance evaluation criteria, and eight different prediction models for eight datasets are used, including AR model, weighted hidden Markov autoregressive model (WHMAR) [41], RBF neural network model (RBFNN), OPELM, Volterra, pruned lazy learning model (LLpruned) [43], least squares support vector machines (LSSVM) [44], and the VKOPP model proposed in this paper. Moreover, all algorithms in the experiments are given optimal parameters. All experiments are run on the same Microsoft windows XP system with at least 2 GB of memory (no swapping for any of the experiments) and a Pentium Dual-Core E5800 CPU @ 3.20 GHz.

From Table 4, it can be seen that the prediction accuracy of the proposed VKOPP method improved at least one or two orders of magnitude better than the original OPELM and Volterra. Specifically, compared with the strong stochastic Laser, DMT, ED, CATS_B, SN, and RDD sequence, the VKOPP model has better adaptability and thus achieves higher prediction performance. Furthermore, the VKOPP model is always better than, or at least as good as, the other prediction models, with an improvement in the MSE and NRMSE of the results. Obviously, the one-step prediction performance of the VKOPP model is satisfactory for application.

For the long-term prediction, as shown in Table 5, the VKOPP model prediction accuracy is also better than that of the original OPELM and Volterra, and for some data sets, the prediction errors of the original OPELM and Volterra are infinite due to the reason that they are not convergent. For the strong stochastic sequences, this shows that compared with several conventional nonlinear models (such as RBFNN, OPELM, and LSSVM), the VKOPP model can achieve higher multistep prediction accuracy to the time series, but compared with the AR model, the advantage is not very obvious.

In view of the MG_S dataset, which contains singular points, the results of the VKOPP model presented in Table 4 and Table 5 have higher prediction precision than the other algorithms. Indeed, this shows that the VKOPP model has high robustness.

In summary, the experimental results have shown that in both single-step and multistep prediction, the VKOPP model proposed in this paper can achieve higher prediction accuracy for the different types of random sequences.

### 6.2. IGBT’s RUL Prediction Results and Analysis

**Figure 8:**
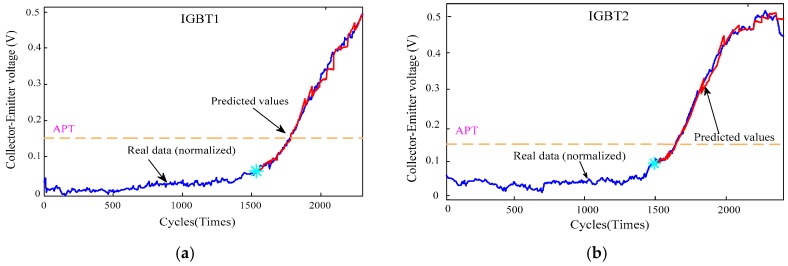

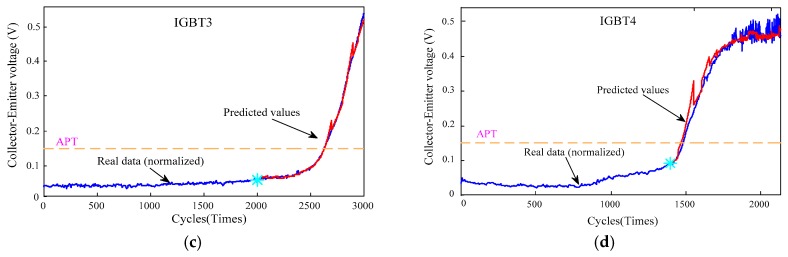
The 50-step-ahead prediction result: “true” (blue line), and predicted by VKOPP model (red line) (In each IGBT experimental dataset, two-thirds are taken for the training set). (**a**) The prediction result of the IGBT1; (**b**) The prediction result of the IGBT2; (**c**) The prediction result of the IGBT3; (**d**) The prediction result of the IGBT4.

From the previous discussion, the experimental circuit has some disturbances during the data collection of the whole failure process, such as instability of the driving waveforms, transport delays of the twisted pairs, stray inductance caused by the load network and PCB circuit board errors, leading to the fact that the collector–emitter saturation voltage of the raw experimental data isnot equal to the typical value provided by the device manual and is mixed with noisy data and bad points.Therefore, before performing the failure prediction of the IGBT, the original data need to be pre-processed, including getting rid of bad points, signal denosing, normalization and dimensional reduction. In order to facilitate a more accurate prediction of IGBT RUL, we utilize four methods to compress and convert the raw data extracted to obtain the best features in a low-dimensional space. First, use the 3σ criterion to exclude the bad points. Second, obtain the average of the collection data for each cycle, which was seen as characteristic of the cycle. Then, execute normalization to align all probability distributions of the average. Finally, the data are filtered by wavelet processing. Figure 8 shows the processed data and the prediction results of the VKOPP model for the four IGBTs.

Figure 8 (blue line) shows the saturation voltage decrease V_CE_ fade trends with four groups of experiments labeled IGBT1, IGBT2, IGBT3 and IGBT4 from the above temperature cycling test, where APT stands for the acceptable performance threshold. For many applications, when the measured V_CE_ deviates ±15% fromits “normal” reference value, this IGBT is considered as seriously “degraded” and should be replaced [28]. Therefore, the value of APT in our paper is 0.15, which is 15% of the rated value.

**Figure 9:**
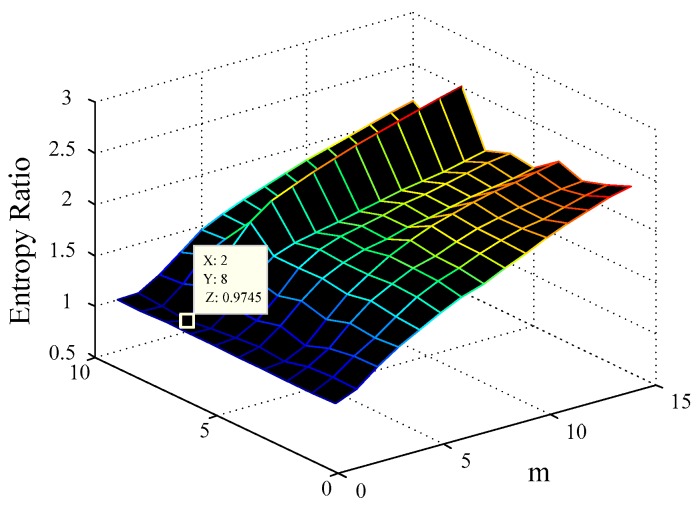
IGBT experimental data (IGBT3) entropy ratio diagram.

For each IGBT experimental dataset, the minimal differential entropy ratio (ER) is utilized to optimize the embedding dimension m and delay time τ at the same time e.g., the ER diagram of IGBT4 and IGBT3 experimental data are shown in Figure 5 and Figure 9, respectively. In addition, selecting 100 nearest neighbors with Volterra truncation order p=1, the VKOPP method is used with all possible kernels—linear, sigmoid, and Gaussian—using a maximum of 100 neurons.

When the parameter design of the VKOPP algorithm is completed, the forecasting process will be carried out (the specific steps are shown in Section 4 of this paper). To better validate the prediction performance of our proposed prognostic approach, three experiments with different experimental conditions are conducted: (1) use the different numbers of measurement data points as the training data to predict the RUL under the same forecast steps; (2) take two-thirds in each IGBT experimental dataset for the training set to predict the RUL under the different forecast steps; (3) compare the VKOPP algorithm with other typical machine learning algorithms to predict the IGBT RUL in terms of prediction accuracy and time consumption.

In the first experiment, from the test results in Table 6, it is appreciated that the VKOPP method has good approximation, and the prediction errors are under 1.5% for training cycles from the minority to the majority under 50-step-ahead forecasting. In addition, a small number of training cycles can also obtain a good prediction result using the proposed prognostic approach.

**Figure 10:**
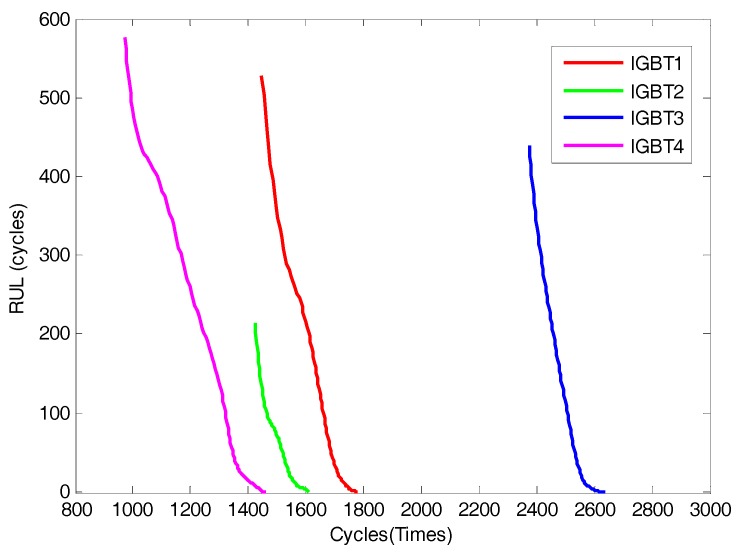
The predicted RULs of the four IGBTs.

In the second experiment, the life prediction error results with prediction steps 1, 10, 50 and 100 for each IGBT experimental dataset are shown in Table 7, and Figure 10 shows the predicted RULs of each IGBT. As shown in Table 7, with increasing number of prediction steps, the error increases gradually, but not very obviously. This illustrates that in both single-step and multistep prediction, the VKOPP model proposed in this paper can achieve higher prediction accuracy for each IGBT experimental dataset. In addition, in Figure 8, experimental results show that even with 50-step-ahead prediction, not only is the data trend in the prediction similar to that of the actual dataset but the predicted life is also close to the actual acceptable performance threshold.

**Figure 11:**
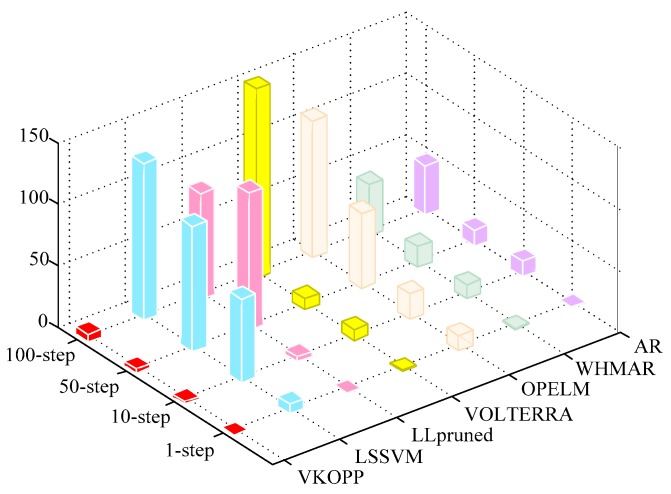
The three-dimensional histogram of the life prediction error results for different methodologies.

In the last experiment, to further estimate the prediction effect, a comparative study given by other typical machine learning algorithms was presented (shown in Table 8 and Table 9 and Figure 11).

The algorithms involved in the Table 8 and Table 9 and Figure 11 are briefly described in Section 5. For the IGBT3 experimental dataset, two-thirds (2000 samples) are taken for the training set. In addition, all experiments have been run on the same Microsoft Windows XP system with at least 2 GB of memory (no swapping for any of the experiments) and a Pentium(R) Dual-Core E5800 CPU @ 3.20 GHz.

(1) *Prediction Accuracy:* With the life prediction results of different prediction steps for the proposed and conventional prediction methodologies tested from Table 8 and Figure 11, the proposed prognostic approach can predict the life of IGBT modules with less error than other algorithms, and with increasing number of prediction steps, the advantage is more obvious.
(2) *Time-consumption:* Table 9 reports the time consumption of 50-step-ahead prediction for experimental dataset IGBT3. The results of Table 9 show the interesting fact that the proposed VKOPP algorithm is computationally efficient, within approximately 1.747 s, to predict the RUL when 2000 samples are used as the training data. Furthermore, compared with some typical machine learning algorithms (i.e., WHMAR, OPELM, LLpruned, and LSSVM), the VKOPP algorithm has an obvious advantage in computational time.

All above experimental results have shown that the proposed prognostic approach can predict the life of IGBT modules with small error. Compared with some typical machine learning algorithms, the model can achieve higher prediction precision. Moreover, the proposed prognostic approach is computationally efficient. Hence, this study illustrates that the VKOPP model strikes a very good compromise between computational speed and prediction accuracy for the RUL prediction of IGBT modules.

## 7. Conclusions

By analyzing the above experimental results, we can draw the following conclusions: (1) The VKOPP method achieves good approximation of IGBT RUL, and the prediction errors are low for training cycles from the minority to the majority under the same forecast steps. The prediction result can also be good with a small number of training cycles. (2) For both single-step and multistep prediction, the VKOPP model proposed in this paper can achieve higher prediction accuracy for each IGBT experimental dataset. (3) By applying the seven prediction models (i.e., AR, WHMAR, OPELM, Volterra, LLpruned, LSSVM and VKOPP) to IGBT experimental data, the proposed prognostic approach can predict the RUL of IGBT modules with less error than the other six models, and with increasing number of prediction steps, the advantage is more obvious. (4) The proposed VKOPP algorithm is computationally efficient. However, compared with some other algorithms, the advantage in computational time is not very obvious, which will be the focus of future research work on the VKOPP model.

## Figures and Tables

**Table 1:** Typical failure mechanisms and external characteristic parameters.

Typical Failure Mechanisms	External Characteristic Parameters
Thermal stress (solder fatigue)	Junction-case thermal resistance
Electrical Stress (wire-bond)	Gate voltage
Thermal stress (wire-bond, solder fatigue)	Turn-off time
Thermal stress/Electrical Stress	Saturation voltage

**Table 2:** Pros and cons of the four characteristic parameters.

	Junction-Case Thermal Resistance	Gate Voltage	Turn-Off Time	Saturation Voltage
Pros	Direct response module aging condition	Basically unaffected by the device working point	Direct response status change	Simple measurement, high accuracy
Cons	The junction temperature is essential for calculating thermal resistance, but it is difficult to access. Direct measurement uses a sensor close to the junction, but this is intrusive and the accuracy is affected by sensor positioning and thermal inertia.	The real-time measurement of high requirements, vulnerable to the influence of the stray capacitance of the circuit.	Request the sensor response time as the nanosecond level, project cost is too high	Affected by the case temperature and collector current

**Table 3:** Frequency and temperature swing of cycle test.

Number	Frequency (Hz)	Swing ΔT (°C)
IGBT1	1 k	100
IGBT2	5 k	100
IGBT3	1 k	50
IGBT4	5 k	50

**Table 4:** One-step-ahead prediction: MSE in the top line (NRMSE in the bottom line) for all eight methodologies for eight datasets. (×10^−^^3^).

	*MG*	*Laser*	*DMT*	*ED*	*CATS_B*	*SN*	*RDD*	*MG_S*
AR	0.00	11.00	9.70	0.88	0.46	2.90	1.80	0.14
	5.60	400.00	520.00	100.00	110.00	290.00	260.00	57.00
WHMAR	0.00	10.00	10.00	0.91	0.45	2.90	1.90	130.00
	3.80	380.00	530.00	100.00	110.00	290.00	260.00	1800.00
RBFNN	0.00	340.00	71.00	430.00	3100.00	690.00	13,000	0.68
	3.60	2200.00	1400.00	2300.00	9100.00	4500.00	22,000	120.00
OPELM	0.00	7100.00	1100.00	810,000.00	130,000.00	190,000.00	270.00	0.16
	5.60	430.00	640.00	100.00	110.00	310.00	250.00	56.00
Volterra	0.00	33.00	21.00	3.40	2.50	16.00	2800.00	0.11
	11.00	680.00	770.00	200.00	260.00	670.00	1000.00	51.00
LLpruned	0.00	1.60	11.00	0.93	0.74	3.90	3.00	0.03
	8.30	150.00	560.00	110.00	140.00	340.00	340.00	26.00
LSSVM	0.00	13.00	21.00	24.00	140.00	6.00	7.70	0.07
	8.90	420.00	760.00	540.00	1900.00	420.00	530.00	40.00
VKOPP	0.00	0.76	9.40	0.78	0.48	2.80	1.60	0.02
	0.39	100.00	510.00	97.00	110.00	280.00	240.00	23.00

**Table 5:** Ten-step-ahead prediction: MSE in the top line (NRMSE in the bottom line) for all eight methodologies for eight datasets. (×10^−^^3^).

	*MG*	*Laser*	*DMT*	*ED*	*CATS_B*	*SN*	*RDD*	*MG_S*
AR	3.70	20.00	15.00	9.90	2.00	5.30	2.10	4.40
	290.00	540.00	640.00	350.00	230.00	390.00	280.00	320.00
WHMAR	5.30	17.00	15.00	10.00	2.20	5.30	2.00	11,000.00
	350.00	490.00	660.00	350.00	240.00	390.00	270.00	16,000.00
RBFNN	5.90	∞	800,000.00	∞	∞	∞	∞	∞
	370.00	690,000.00	150,000.00	1,800,000.00	∞	6,500,000.00	7,000,000.00	2,300,000.00
OPELM	0.38	2,200,000.00	15,000.00	∞	1,100,000.00	550,000.00	300.00	2.90
	83.00	740.00	790.00	490.00	320.00	520.00	270.00	230.00
Volterra	4.60	67.00	32.00	11.00	360.00	∞	∞	6.80
	320.00	970.00	950.00	370.00	3100.00	∞	∞	390.00
LLpruned	0.03	36.00	21.00	16.00	6.10	9.40	3.80	0.24
	27.00	710.00	770.00	440.00	410.00	520.00	380.00	74.00
LSSVM	0.01	16.00	25.00	40.00	140.00	8.20	17.00	0.10
	11.00	480.00	840.00	690.00	1900.00	490.00	930.00	48.00
VKOPP	0.00	7.50	15.00	4.70	2.30	4.20	1.60	0.09
	1.20	320.00	650.00	230.00	250.00	350.00	240.00	45.00

**Table 6:** The Life prediction error result foreach IGBT experimental dataset at different training cycles (50-step-ahead).

	Cycle Test Conditions	Training Cycles	Life Prediction (Cycles)	Actual Life (Cycles)	Prediction Error (Cycles)	Relative Error (%)
**IGBT1**	*f* = 1 kHz, ΔT = 100 °C	500	1774	1784	10	0.561
		1000	1775		9	0.504
		1506	1776		8	0.448
		1600	1775		9	0.504
**IGBT2**	*f* = 5 kHz, ΔT = 100 °C	500	1614	1615	1	0.062
		1000	1613		2	0.124
		1488	1617		2	0.124
		1550	1613		2	0.124
**IGBT3**	*f* = 1 kHz, ΔT = 50 °C	500	2650	2646	4	0.151
		1000	2649		3	0.113
		2000	2649		3	0.113
		2400	2644		2	0.076
**IGBT4**	*f* = 5 kHz, ΔT = 50 °C	500	1463	1474	11	0.746
		1000	1465		9	0.611
		1348	1461		13	0.882
		1400	1464		10	0.678

**Table 7:** The life prediction error result for each IGBT experimental dataset at different prediction steps.

	Cycle Test Conditions	Prediction Steps (Cycles)	Training Cycles	Life Prediction (Cycles)	Actual Life (Cycles)	Prediction Error (Cycles)	Relative Error (%)
**IGBT1**	*f* = 1 kHz, ΔT = 100 °C	1	1506	1784	1784	0	0
		10		1782		2	0.112
		50		1776		8	0.448
		100		1771		13	0.729
**IGBT2**	*f* = 5 kHz, ΔT = 100 °C	1	1488	1616	1615	1	0.062
		10		1612		3	0.186
		50		1617		2	0.124
		100		1620		5	0.309
**IGBT3**	*f* = 1 kHz, ΔT = 50 °C	1	2000	2646	2646	0	0
		10		2648		2	0.076
		50		2649		3	0.113
		100		2652		6	0.227
**IGBT4**	*f* = 5 kHz, ΔT = 50 °C	1	1348	1473	1474	1	0.068
		10		1474		0	0
		50		1461		13	0.882
		100		1457		17	1.153

**Table 8:** The life prediction error result for different methodologies.

	Life Prediction Error (IGBT3 Experimental Dataset)			
	1-Step	10-Step	50-Step	100-Step
AR	1	13	13	40
WHMAR	1	11	17	42
OPELM	12	21	61	111
Volterra	1	9	9	154
LLpruned	0	4	112	86
LSSVM	8	68	102	128
VKOPP	0	2	3	6

**Table 9:** The time consumption of several prediction algorithms for experimental dataset of IGBT3.

	Time Consumption (50-Step-Ahead) (s)
AR	0.6
WHMAR	37.8
OPELM	4.5
Volterra	0.799
LLpruned	90.23
LSSVM	223
VKOPP	1.747
